# Collagen Supplementation for Joint Health: The Link between Composition and Scientific Knowledge

**DOI:** 10.3390/nu15061332

**Published:** 2023-03-08

**Authors:** Daniel Martínez-Puig, Ester Costa-Larrión, Nuria Rubio-Rodríguez, Patricia Gálvez-Martín

**Affiliations:** R&D Bioiberica S.A.U., E-08389 Palafolls, Spain

**Keywords:** native collagen, hydrolyzed collagen, nutritional supplement, joint health, osteoarthritis

## Abstract

Osteoarthritis (OA) is the most common joint disease, generating pain, disability, and socioeconomic costs worldwide. Currently there are no approved disease-modifying drugs for OA, and safety concerns have been identified with the chronic use of symptomatic drugs. In this context, nutritional supplements and nutraceuticals have emerged as potential alternatives. Among them, collagen is being a focus of particular interest, but under the same term different types of collagens coexist with different structures, compositions, and origins, leading to different properties and potential effects. The aim of this narrative review is to generally describe the main types of collagens currently available in marketplace, focusing on those related to joint health, describing their mechanism of action, preclinical, and clinical evidence. Native and hydrolyzed collagen are the most studied collagen types for joint health. Native collagen has a specific immune-mediated mechanism that requires the recognition of its epitopes to inhibit inflammation and tissue catabolism at articular level. Hydrolyzed collagen may contain biologically active peptides that are able to reach joint tissues and exert chondroprotective effects. Although there are preclinical and clinical studies showing the safety and efficacy of food ingredients containing both types of collagens, available research suggests a clear link between collagen chemical structure and mechanism of action.

## 1. Introduction

Osteoarthritis (OA) is becoming one of the most common joint conditions due to the increase in life expectancy [1], representing nowadays a major socioeconomic and public health issue [2]. OA is characterized by the inflammation and progressive destruction of articular cartilage, affecting any joint although is more prevalent in knee, hip, spine, and interphalangeal joints causing pain, functional limitations, and reducing quality of life [3,4].

There are different treatment options for OA including pharmacological and non-pharmacological approaches. The most used pharmacological treatments have been analgesics (paracetamol) and non-steroidal anti-inflammatory drugs (NSAIDS) [5]. However, safety concerns associated with their long-term administration have limited its use, particularly in those patients with comorbidities [6,7]. For this reason, efforts have been focused on finding alternative treatments to improve clinical symptoms with better safety profile and tolerability, known as Symptomatic Slow Action Drugs for OA (SYSADOAs) such as glucosamine (GS) and chondroitin sulfate (CS) which are currently the most used SYSADOAs [8]. However, non-pharmacological treatments based on the intake of collagen as nutritional supplement have been positioned as an emerging focus of interest to support a preventive or therapeutic effect in patients with OA [4].

Collagen is the most abundant protein in the extracellular matrix (ECM) and connective tissues of vertebrates, being more abundant in mammalian species [9]. The typical structural element of collagen is a rod-like triple-helical domain. Based on their structure, supramolecular organization, and functional features, 28 types of collagens have been described [10]. The obtaining of collagen mostly relies on extraction from animal-derived collagen rich tissues such as cartilage, skin, and bones. Depending on the manufacturing process, different collagen-derived products can be obtained with totally different structure, composition, and properties such as: undenatured native collagen (insoluble) or soluble native collagen, both of which maintain the triple helix structure; gelatin (denatured collagen), and hydrolyzed collagen (peptides/amino acids) which in turn can be produced with different degrees of hydrolysis [11].

The molecular structure of orally administrated collagen determines its mechanism of action for joint heath. Initially, it was postulated that collagen supplementation could promote the synthesis of connective tissue, especially cartilage ECM [12], mainly because collagen represents its major component [13]. In fact, it has been demonstrated that certain peptides from hydrolyzed collagen are absorbed and accumulated in the cartilage [14]. In addition, animal models of OA have obtained promising results in terms of preservation of cartilage structure as a result of long-term ingestion of hydrolyzed collagen [15,16]. Regarding native collagen (both soluble or insoluble), the most studied is type II, which was evaluated initially in rheumatoid arthritis [17] and afterwards in OA [18]. It has been reported that native type II collagen elicits an immune-mediated response called oral tolerance [19], a totally different mechanism compared with the one described for hydrolyzed collagens. According to this mechanism of action, native type II collagen would reduce autoimmune reactions against endogenous collagen at articular cartilage level.

Collagen has been extensively studied for years. Only in the past decade, more than 20,000 papers have been published about different facets of collagen describing its molecular structure of triple helix, its natural occurrence, its physicochemical properties and biological functions, extraction methods, or its new applications. However, as under the same term different types of collagens coexist, there is certain confusion about the therapeutic potential of each one, depending on its structure and composition.

Thus, the aim of the present review is to define the different types of collagens from a structural point of view, review the proposed mechanisms of action associated with each form for oral use, and compile its preclinical and clinical evidence for joint health.

## 2. Understanding Collagen World

Collagen is an ancient term coined to name the natural adhesive obtained by cooking animal bones. It is though that derives from Greek κόλλα (kólla or “glue”) and -γενής (-genḗs or “producing”). From a scientific standpoint, collagen is now defined as a large family of structural proteins found in the ECM of animal tissues that are distinguished for containing one or more domains with a unique triple helical structure [20]. Triple helical conformation of collagen was first described in the 1950s according to X-ray diffraction pattern of collagen fibers found in skin [21]. This structure comprises three left-handed polypeptide chains (so-called collagen α-chains) that are coiled into a right-hand helical structure. Collagen polypeptide chains are distinguished for having a repeated specific unit (Gly-X-Y) in which Gly is glycine (the smallest amino acid in nature); and X and Y are frequently proline (Pro) and hydroxyproline (Hyp). Due to this specific motif, several molecular interactions take place in the collagen triple helix leading a unique close packaging along a central axis [22].

Knowledge about collagen has been exponentially increased in the past decades. Whereas scientific community in the 1970s talked about only four types of genetically distinct collagens [23], at present it is generally accepted that collagen superfamily comprises up to 28 members that differ from each other in molecular composition as well as in their supramolecular organization within the ECM [24,25,26,27]. The occurrence of an additional collagen type, called collagen XXIX, has been also claimed by some authors [28,29], although, given that COL29A1 gene was shown to be identical to the COL6A5 gene and that the a1(XXIX) chain corresponds to the a5(VI) [30], collagen XXIX is not generally accepted as a genetically distinct family [24].

Depending on their supramolecular organization, collagen types can be additionally assorted in different families such as fibril-forming collagens, basement membrane collagens or microfibrillar collagens, among others [10,25].

Fibril-forming collagens are by far the most abundant in nature. They comprise type I, type II, type III, type V, and XI. All these collagen types have in common the ability to assemble into a quarter-staggered fibril-array. Withing this group, type I and type II occur to a greater extend. Type I collagens are heterotrimeric molecules, [α1 (I)]_2_ α2 (I)], comprising two identical polypeptide chains [α1 (I)] and a different one, [α2 (I)]. They represent more than 90% of organic matter in bone, dermis, tendon, ligaments, and cornea. Type II collagens are homotrimer molecules, [α1 (II)]_3_, comprising three identical polypeptide chains. They account for about 80% of total collagen in cartilage but also occur in other tissues such as vitreous body or cornea among others [10].

It is worth noting that other fibrous proteins such as elastin have been found in ECM along with fibril-forming collagen [31]. However, collagen fibers and elastin differ in both chemical composition [32] and supramolecular organization [33]. Conversely, it has been reported that certain plasma and cell surface proteins (i.e., collectins, C1q, or ficolin) exhibit collagen triple helix domains as well, but they have been excluded from collagen family as they do not occur in the ECM [34].

Biosynthesis of collagen has been observed in mesenchymal cell family (fibroblasts, chondroblasts or osteoblasts) as well as in other cell lines such as epithelial cells [23]. It in-volves an intricate multi-step process that starts with the transcription of collagen genes, follows up with several intracellular reactions, and finishes with extracellular processing. Intracellular reactions comprise ribosomal protein synthesis (translation), post-translational modifications, assembly of the three α-chains into trimeric collagen monomers (also known as procollagen) and secretion to ECM; whereas extracellular processing comprises complex cross-linking reactions that convert procollagen into a supramolecular structure [30].

No evidence has been found so far about the presence of collagen in other living beings such plants or unicellular microorganisms. Structural role of collagen in those lower phyla seems to be replaced by other compounds, mainly polysaccharides or protein–polysaccharide complex [35].

Collagen has been historically valorized in several industrial fields. Given that it is an edible protein, a large number of industrial applications have been focused on food industry, where collagen commonly plays different food technological functions such as emulsifier, film-forming material, gelling agent, or stabilizer among others [36]. Moreover, due to its mechanical properties and biodegradability, collagen is also considered an ideal material for many biomedical applications such as skin substitute, scaffold manufacturing for tissue engineering (bone, tendon, or cartilage), neural repair or drug delivery systems including hydrogels, granules, microcapsules, or microspheres [37].

It is worth mentioning that not all the collagens described in the literature for a commercial application are equal, but there is plenty of variety in terms of amino acid composition or molecular structure as well as in term of physicochemical properties or biological activities. Such variety is not only related to raw material but also to manufacturing process.

The great majority of collagens currently found in the marketplace are obtained from animal-origin raw materials. Skins, tendons, bones, and hides are the most typical, which basically comprise connective tissue and thus are an abundant source of type I collagen [38]. In addition, cartilages are used for production of type II collagen [39,40] and, in a minor extend, eggshell membranes are proposed as a natural raw material to obtain type I, V, and X collagen [41]. Traditional animal species chosen for collagen production are porcine and bovine, although poultry [42] and fish [43] are becoming more popular to over-come religious limitations of porcine collagen and concerns about bovine zoonotic diseases.

**Figure 1:**
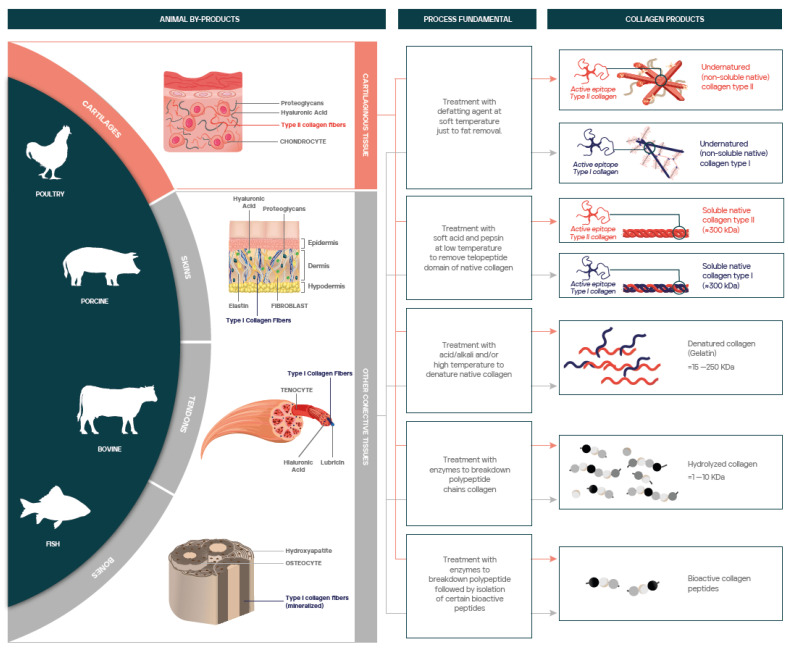
Overview of main collagen products that can be obtained from natural sources (animal by-products) according to their origin (animal and tissue) and manufacturing process. Hydrolyzed collagens can be obtained from cartilage or other connective tissues through an enzymatic treatment to breakdown polypeptide chains. Undenatured (non-soluble) or soluble native collagen type II can be only obtained from cartilage, through soft temperature processes to maintain the triple helix structure intact.

Manufacturing processes to obtain collagen from a natural source usually involve different extraction and purification techniques, which shape the main features of the final product as physicochemical properties or biological activities. As a result, different collagen products have been described, such as “insoluble undenatured native collagens”, “soluble native collagens”, “denatured collagens”, “collagen hydrolysates”, and “collagen peptides” (Figure 1; Table 1).

Insoluble undenatured native collagens are distinguished for maintaining intact the triple helix structure (native collagen), which are resistant to proteases, along with the covalent crosslinking that naturally occurs in animal tissues, specially at the terminal non-helical domains. To achieve those features, the production process must avoid using high temperatures and denaturation or solubilization agents [44]. Insoluble undenatured native collagens are characterized for being non-soluble in water as well as for exhibiting antigenic sites (epitopes), which depend on collagen type and consequently on the raw material used. The preservation of certain collagen epitopes, associated to fibrillar structure, has been shown to be involved in immune-mediated effects [45,46]. Hence, if the raw material is mostly composed by cartilaginous tissue, such as chicken sternum, the resulting product is known as insoluble undenatured type II collagen [39]. As further described in Section 3.1, this product exhibits specific epitopes related to type II collagen which promote a reduction in inflammation related to OA when taken orally.

Procedures for obtaining insoluble collagens from skin, tendon, bone, or heart [44,47,48], have been also found in the literature. To the best of our knowledge, no industrial or medical applications have been described about these products.

Soluble native collagens are characterized for having intact the triple helix but a fewer crosslinking than the insoluble products. Thereby, from a chemical standpoint, collagen in these products only maintains its tertiary structure (triple helix) whose molecular weight is 300 kDa on average. To match those features, the production process is carried out at low temperatures but includes the addition of a solubilization agent able to destabilize selectively the covalent bonds in natural crosslinking. A wide range of solubilization agents have been reported in the literature [43], leading to different “soluble collagens” with specific physicochemical properties. Among them, “acid solubilized collagen” (ASC) and “pepsin solubilized collagen” (PSC) are the most commonly found in the market. Soluble native collagen can also be distinguished by its collagen type, which can be identified according to its antigenic sites (epitopes).

Denatured collagen, also known as “gelatin”, are differentiated for having lost the triple helix structure because of the action of temperature and/or a denaturing agent. Therefore, they are not composed by collagen molecules but by a random mixture of polypeptide chains that usually range from 15 to 250 kDa [14] and that are characterized by a high content in Hyp. Depending on the raw material and extraction procedure, gelatins can differ in composition but also in solubility behavior and rheological properties, being gel strength and thermal stability the main quality attributes. Common gelatins in marketplace are “type A” (isoelectric point at pH ≈ 8–9) and “type B” (isoelectric point at pH ≈ 4–5), which are extracted with acids and alkalis, respectively [38]. Gelatins are widely used in food industry, either as a gelling agent, emulsifier, foamer, or coating material for encapsulation [36,38]. Unlike native collagens, gelatins cannot be labeled as a specific collagen type since this factor is inherent to the triple helix structure. For the same reason, gelatins are not immunogenic since active epitopes related to the collagen triple helix have been lost in the denaturation step.

Collagen hydrolysates are distinguished for having lost the triple helix structure as well but, unlike to gelatins, they are subjected to a chemical or enzymatic hydrolysis process to breakdown polypeptide chains [36]. As a result, collagen hydrolysates are composed by a mixture of amino acids and peptides which composition depends on both collagen source and hydrolysis method. For instance, collagen hydrolysates prepared by enzymatic hydrolysis with pepsin, alcalase or papain, exhibit a molecular weight distribution ranging from 1 kDa to 10 kDa [49].

As described in gelatins, collagen hydrolysates are not immunogenic and cannot be labeled as a specific collagen type. However, conversely to gelatins, they may include some specific peptides, called “collagen peptides”, that exhibit different bioactive properties when they are isolated by any purification process. So far, a huge amount of different collagen peptides has been described in the literature showing different in vitro activities such as antioxidant activity, ACE-I inhibitory activity, or DPP-IV inhibitory activity [36]. As far as we know, in most of the commercial products for joint mobility, collagen peptides are not isolated from hydrolyzed collagen but mixed with other peptides and amino acids resulting from collagen hydrolysis.

Besides collagens obtained from natural animal sources, some attempts have been made to produce “non-animal collagens” either by chemical synthesis or by biotechnological means. Synthetic collagen-related peptides (CRPs) were first designed and assembled by in the late 1960s [50]. Despite the latest developments to mimic the structure of collagen triple helix as well as the fibril formation [22], synthetic collagens are still exceedingly simplified structures compared to natural collagens [39]. Recombinant DNA technology was first developed in the 1990s as an alternative to synthetic methods to obtain non-animal collagens. Since then, various expression systems have been described in the literature to produce native-like recombinant collagens and their fragments. However, large-scale production of collagen by genetic engineering is still highly limited and only a few yeast cells and transgenic plants are currently implemented to produce recombinant collagens for specific biomedical applications [51].

In addition, the term “vegan collagen” or “vegan collagen builder” has been also recently introduced in functional food market to refer to certain products that are basically composed of a blend of ingredients including plant extracts, amino acids, vitamins, and minerals. Even though some studies have evidenced that collagen biosynthesis is mediated by certain micronutrients contained in these ingredients, such as vitamin C [52], copper or zinc [53,54], it is worth noting that, to the best of our knowledge, no product labelled as “vegan collagen” contains actual collagen in their ingredient list.

## 3. Mechanism of Action in Joint Health

Based on the molecular structure of collagen, different mechanisms of action have been described for its use as ingredient in the manufacturing of food supplements. Native collagen (insoluble or soluble) is resistant to proteinases and therefore it is not digested across the gastrointestinal tract [44], being able to maintain the triple helix structure and enhance joint health by means of the oral tolerance mechanism [19]. In contrast, both gelatin and hydrolyzed collagens lack the triple helix and, consequently, the oral tolerance mechanism of action is lost. Gelatin is likely the most frequent form in the market, but no biological function for joint health has been described. However, it has excellent physical and mechanical properties such as low solubility and adequate handling, mainly due to the fact that it is composed of a mixture of peptides with different molecular weights [55]. Thus, gelatin is widely used in the manufacturing of different food systems such capsules and films [56].

Finally, hydrolyzed collagens are composed of amino acids and peptides of varying lengths (including dipeptides and tripeptides) which resist the intracellular hydrolysis process avoiding their degradation by peptidases and systemic hydrolytic enzymes. Thus, the peptides from hydrolyzed collagens have a high bioavailability allowing them to reach the bloodstream, accumulating in the cartilage tissue and inducing the synthesis of cartilage ECM, by stimulating the chondrocytes [57].

The differences in the mechanisms of action described for native and hydrolyzed collagens, could even justify theoretically a potential combination of both types of collagens to explore the complementary effects.

### 3.1. Native Collagen

In its native form, collagen has a specific immune mediated mechanism of action known as oral tolerance. Oral tolerance has been defined as the active suppression of specific immune responses to antigens first encountered in the gastrointestinal tract [58]. It represents an immune-mediated mechanism responsible of avoiding immune responses against harmless antigens, such as food proteins or commensal organisms.

The basic description of oral tolerance has been exhaustively reviewed [59,60,61]. The process is initiated in the gut-associated lymphoid tissue (GALT) but has an impact on the systemic immunity [61]. Briefly, luminal antigens are captured by antigen-presenting cells, which migrate into gut-draining mesenteric lymph nodes where they initiate activation and differentiation of effector or regulatory T cells (Tregs) [62]. These antigen-specific regulatory cells control immune response inducing the secretion of down-modulatory cytokines such as TGF-b, IL-10, and IL-4, while decreasing pro-inflammatory cytokines [61,63,64].

The mechanism of oral tolerance has been largely demonstrated in various animal models of autoimmune diseases, such as in collagen-induced arthritis [19]. In this model of rheumatoid arthritis, the autoimmune response against cartilage type-II collagen, is induced by injecting type II collagen and Freund’s adjuvant to susceptible mice (i.e., DBA/1) [65].

The oral tolerance to type II collagen was first demonstrated by Nagler-Anderson in 1986 [66]. Interestingly, in this earlier work, the oral administration of native type-II collagen but not denatured type-II collagen, reduced the autoimmune response, showing that the triple-chain collagen structure is required to elicit the oral tolerance response. Additionally, the efficacy of native type-II collagen to control articular inflammation has been also demonstrated in other animal models of rheumatoid arthritis such as pristane induced arthritis [67] and adjuvant arthritis [68]. In this latter model, oral administration of native type II collagen suppressed the development of arthritis in Lewis rats, and this suppression could be adoptively transferred by T cells from native-type II collagen-fed animals.

The mechanism of oral tolerance has been evaluated with the aim of developing therapeutic alternatives for autoimmune diseases. This is not the case for OA, although accumulating evidence suggests that deregulations of the immune response have an impact on disease pathogenesis along with other mechanical and biochemical factors [69,70].

Historically, OA has been defined as a simple degradation of joint cartilage associated to the ageing process [71]. Afterwards it was recognized that the disease affects not only the cartilage but the full joint structure, and that structural changes are driven not only by mechanical factors but also by inflammation [72]. Inflammation has been shown to be triggered and/or amplified by an immune response against autoantigens released by the degradation of joint tissues [69,70].

Type II collagen (the main protein of the articular cartilage) has been shown to be a potential source of autoantigens in OA [73,74,75]. In consequence, oral tolerance against type-II collagen could theoretically have a positive impact to control inflammation in OA. In fact, the efficacy of oral administration of low doses of native type-II collagen has been demonstrated in animal models of OA such as the rat model of OA induced by monoiodoactetate (MIA) [76]. In this model, oral administration of chicken native type II collagen (1–10 mg/kg) reduced articular pain, decreased plasmatic concentration of inflammatory cytokines (TNFa, IL-1b), and reduced cartilage degradation as shown by a reduction in the plasmatic levels of C2C.

**Figure 2:**
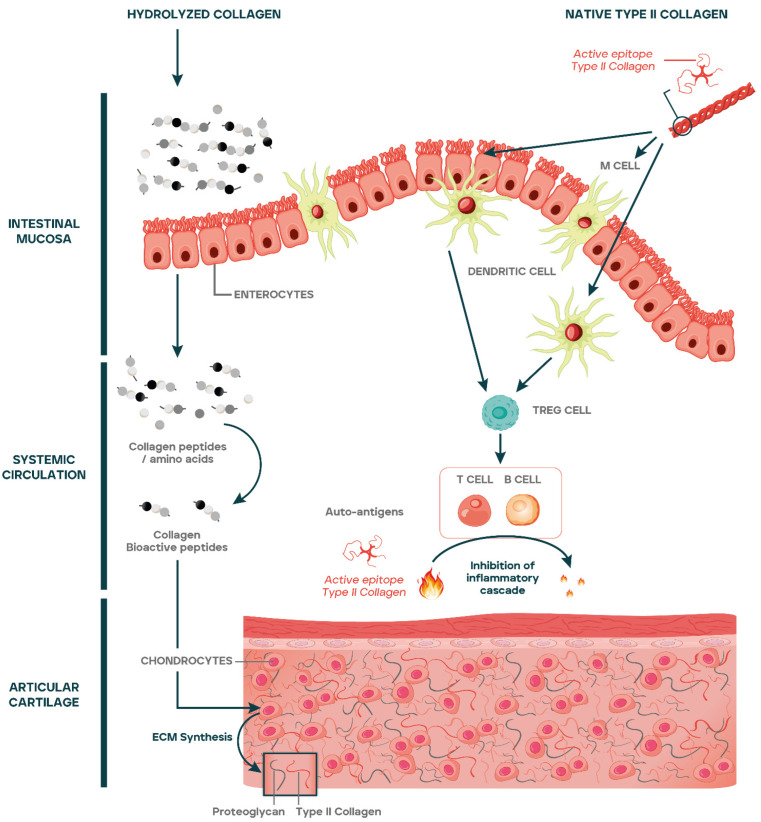
Amino acids and peptides from hydrolyzed collagens are absorbed reaching the systemic circulation and articular cartilage, stimulating the synthesis of ECM macromolecules and chondrogenic differentiation. Native type II collagen epitopes cross the gut lumen by different mechanisms including uptake by M cells, transport across enterocytes, or through tight junctions. The epitopes are passed on to dendritic cells that once activated favor the differentiation of regulatory T cells (Tregs) at Peyer’s Patches or at mesenteric lymph nodes. Tregs exit from lymph nodes to systemic circulation through the efferent lymph and reach articular cartilage. At articular cartilage, Tregs inhibit the inflammatory cascade caused by the release of autoantigens generated as a consequence of cartilage catabolism.

The mechanism of action of native type II collagen in OA as compared to hydrolyzed collagens has been summarized in Figure 2.

### 3.2. Hydrolyzed Collagens

Bioavailability of amino acids and peptides from hydrolyzed collagens is a pivotal aspect to explain the effects of the product at the articular level. In general terms, it has been demonstrated that peptides resistant to intracellular hydrolysis have lower molecular weight and show higher intestinal absorption [77,78]. Levels of collagen-derived dipeptides such as Pro-Hyp and tripeptides such as Pro-Hyp-Gly have been detected in systemic blood after an hour of being ingested [79]. Then, these peptides reach the joint tissues such as cartilage where they accumulate [14].

Once in the cartilage, in vitro studies have demonstrated that collagen peptides exert different biological effects that may be dependent on the peptide and amino acid profile of the hydrolyzed collagen [80]. Thus, different studies have shown that collagen peptides stimulate the synthesis of ECM macromolecules such as proteoglycans and type II collagen [81], induce chondrogenic proliferation and differentiation [82], increase the activity of osteoblasts [83], and decrease the activity of osteoclasts [14]. All these effects suggest that hydrolyzed collagen may promote cartilage repair by acting as a chondroprotector in OA [4].

However, in vivo confirmation of the mechanisms described using the in vitro systems has led to mixed results. Comblain et al. [84] studied the effect of a formulation containing hydrolyzed collagen, curcuminoids, and green tea extract in dogs with OA. Although improvements in pain were reported, no effect on biomarkers of cartilage catabolism (Coll2-1 and Coll2-1 NO_2_) was detected. Contrarily, Dar et al. detected a chondroprotective effect consisting in MMP-13 and apoptosis reduction as a result of the administration of HC to mice with induced meniscal-ligamentous injury [85]. Lee et al. [86] also demonstrated chondroprotective effects of a low-molecular weight collagen peptide from fish origin in an anterior cruciate ligament transection rabbit model.

Differences among different hydrolyzed collagens were evaluated by Schadow et al. [87] in a study in human OA cartilage explants in which three different products were analyzed. None of the tested products stimulated the biosynthesis of type II collagen. In addition, great differences were detected on biological activity of each hydrolyzed collagen, showing that the effects of a particular product cannot be extrapolated to another obtained through a different process and consequently containing a different peptide composition. In a subsequent study, Simons et al. [88] showed that differences in peptide profiles could be detected even in different batches of the same hydrolyzed collagen [88] which could potentially lead to different activities.

According to these findings, it seems that linking the bioavailability and biologic effects to a standardized peptide composition would be required to ensure that the final collagen product is effective for OA patients.

## 4. Clinical Evidence

Up to date, several clinical trials regarding the use of collagen as a food supplement for joint health have been published. Most of the studies have evaluated the therapeutic potential of either native type II collagen [89,90,91,92,93,94,95,96,97,98] or hydrolyzed collagens [99,100,101,102,103,104,105,106,107] in patients with OA. However, both types of collagens have been also tested in non-osteoarthritic individuals suffering joint discomfort [108,109,110,111,112,113].

There are also some trials evaluating the use of native type II collagen for rheumatoid arthritis. These trials represented the translation from the initial studies with animal models demonstrating the oral tolerance mechanism. The studies obtained mixed results [17]. Although some positive results were observed in phase II trials, no effect was detected in phase III [114]. The explanation for this lack of efficacy has not been fully elucidated but the gut microbiota as well as the overall inflammatory situation of the patient, are supposed to play a critical role [17].

In general, the studies evaluating the use of native type II collagen for OA have reported positive results in terms of pain relief and joint function improvement, although huge differences exist in study designs and methodologies (Table 2). In a randomized double-blind controlled study, Lugo et al. [92] reported improvements in pain and function after 6 months of the administration of a native type II collagen ingredient (40 mg/day) as compared to a standard treatment with chondroitin sulphate (1200 mg/day) and glucosamine (1500 mg/day). In an observational study, Jain et al. [98] reported improvements of pain and function with the administration of native type II collagen (40 mg/day) combined with a Boswellia extract (1500 mg/day) for a period of 90 days. Except for two initial studies published by Bagchi in 2002 [93] and Scarpellini in 2008 [90], all the following studies used the same dose of 40 mg/day. Moreover, when collagen origin is reported, all studies declare using collagen from a chicken origin.

Fewer studies have evaluated the impact of native type II collagen in cartilage homeostasis. In a retrospective study, Scarpellini et al. showed a reduced progression of cartilage degradation at 6 and 12 months in a group of hand OA patients treated with a combination of native type II collagen, CS and GS [90]. However, in other studies no differences have been detected in serum biomarkers of joint integrity [89,108]. On the other hand, all studies published in non-OA individuals have reported improvements in activity-related joint discomfort and mobility [108,109,110].

In the studies evaluating the use of hydrolyzed collagens for OA, huge variability can be found as different designs, comparators, dosages, administration patterns (either alone or in combination), origins, and study durations are used, making it difficult to draw overall conclusions. Despite huge methodological differences, all studies reported, at least, partially positive results on the evaluated outcomes. Parameters showing most positive results in these trials are related to the self-reported improvement of OA symptomatology including function, quality of life, and pain. Although most of the studies report improvements in pain and function, the daily dose is highly variable. Bernardo et al. [101] in a randomized single-blind study reported improvements in joint pain and function after 6 months of administration of 1.2 g/day of a hydrolyzed collagen ingredient. Using another hydrolyzed collagen, Benito-Ruiz et al. [100] also demonstrated improvements in joint pain and function after 6 months of administration, but with a dose of 10 g/day in a randomized double-blind placebo-controlled study. Besides daily dose, there is also high variability in administration time. Trč and Bohmová [104] in a double-blind study reported improvements in joint pain and function compared to a treatment with GS (1.5 g/day) as a result of the administration of 10 g/day of an hydrolyzed collagen for 3 months, while Kilinc et al. [105] in an observational study reported symptomatic improvements compared to baseline with another hydrolyzed collagen administrated for two weeks at a dose of 720 mg/day followed by two additional weeks at a dose of 360 mg/day.

Besides improvements in pain and function, changes in cartilage degradation have also been detected. In 2011, McAlindon et al. [99] reported an increase in proteoglycan content in knee cartilage after 24 weeks of treatment with 10 g/day of a specific hydrolyzed collagen formulation.

Studies evaluating the effects of hydrolyzed collagens in non-OA patients have obtained mixed results. Two randomized double-blind placebo-controlled studies reported improvements in activity-related joint pain after 6 months of administration of a hydrolyzed collagen from porcine origin at a dose of 10 g/day in one study [112] and 5 g/day in the other [111]. However, in another randomized double-blind placebo-controlled study, no differences in join pain and function were detected after 3 months of administration of 10 g/day of a hydrolyzed collagen from bovine origin [113].

Collagen studies show huge variability among them, but all tested products, collagen types, and dosages seem to deliver positive outcomes (except in one study) and no safety issues were reported.

In summary, the available scientific evidence shows that most tested ingredients seem to deliver positive outcomes although there is huge variability in terms of study designs, effective doses, and minimum treatment periods for each collagen ingredient. When comparing native collagen with hydrolyzed collagen, there is a clear difference in the therapeutic dose, which is smaller in native collagen (40 mg/day) as compared to hydrolyzed collagen (between 5 and 10 g/day). This could have practical implications in terms of galenic development since high daily doses could limit the feasibility to develop certain presentations such as tablets and capsules.

## 5. Conclusions

Collagen has been positioned as an emerging focus of research for articular health. However, the term collagen includes different products with different structures, properties, and mechanisms of action. Native type II collagen has a specific immune-mediated mechanism known as oral tolerance, that inhibits inflammation and tissue catabolism at articular level. Hydrolyzed collagen has been shown to contain biologically active peptides that are able to reach joint tissues and exert chondroprotective effects. There are preclinical and clinical studies showing the safety and efficacy of ingredients containing native type II collagen or hydrolyzed collagen. Nevertheless, available research suggests a clear link between collagen ingredient composition/chemical structure and mechanism of action/efficacy. However further research is required, including well-designed studies, to assess the therapeutic potential of each collagen type and composition for each clinical condition. Novel research would be required to evaluate the potential benefit in populations with risk factors of OA, as well as different OA phenotypes and related disorders, both in terms of symptomatic improvement and progression of cartilage degradation.

## Figures and Tables

**Table 1:** Molecular features of collagen products.

Collagen Product	Collagen Type	Molecular Features		
		Cross Linking	Collagen Triple Hélix	Active Epitopes with Effect in OA
Undenatured native collagen (insoluble)	Type I	✓	✓	✗
	Type II	✓	✓	✓
Soluble native collagen	Type I	✗	✓	✗
	Type II	✗	✓	✓
Gelatin (denatured collagen)	Type I & Type II	✗	✗	✗
Hydrolyzed collagen/collagen peptides				

**Table 2:** Clinical studies on collagen.

Collagen Type	Clinical Condition	Design	Intervention Duration	Daily Dose	Main Results Reported	Reference
Native collagen	Osteoarthritis	Randomized single-blind controlled study	3 months	40 mg	Symptomatic improvement (WOMAC)	[89]
		Observational retrospective study	12 months	2 mg	Reduce progression of cartilage degradation	[90]
		Randomized double-blind controlled study	3 months	40 mg	Symptomatic improvement (WOMAC, VAS)	[91]
		Randomized double-blind placebo-controlled study	6 months	40 mg	Symptomatic improvement (WOMAC)	[92]
		Open-label pilot study	1.5 months	10 mg	Symptomatic improvement (VAS)	[93]
		Non-interventional, prospective real-life study	3 months	40 mg	Symptomatic improvement (WOMAC, VAS)	[94]
		Observational open-label study	4 months	40 mg	Symptomatic improvement (WOMAC, VAS)	[95]
		Randomized double-blind placebo-controlled study	3 months	40 mg	No significant differences vs. controls	[96]
		Prospective controlled study	4 months	40 mg	Symptomatic improvement (WOMAC, VAS)	[97]
		Observational open-label study	3 months	40 mg	Symptomatic improvement (WOMAC, VAS)	[98]
	non-Osteoarthritis	Randomized double-blind placebo-controlled study	4 months	40 mg	Reduced joint discomfort and increased mobility	[108]
		Randomized double-blind placebo-controlled study	6 months	40 mg	Reduced joint discomfort and increased mobility	[109,110]
Hydrolyzed collagen	Osteoarthritis	Randomized double-blind placebo-controlled study	6 months	10 g	Increase of proteoglycan content in knee cartilage	[99]
		Randomized double-blind placebo-controlled study	6 months	10 g	Symptomatic improvement (WOMAC, VAS)	[100]
		Randomized single-blind open-labelled controlled study	6 months	1.2 g	Symptomatic improvement (WOMAC)	[101]
		Randomized double-blind placebo-controlled study	3 months	10 g	Symptomatic improvement (WOMAC, VAS)	[102]
		Randomized double-blind placebo-controlled study	70 days	2 g	Symptomatic improvement (WOMAC, VAS)	[103]
		Randomized double-blind controlled study	3 months	10 g	Symptomatic improvement (WOMAC, VAS)	[104]
		Prospective observational study	1 month	720 mg/360 mg	Symptomatic improvement (WOMAC, VAS)	[105]
		Prospective observational study	6 months	1.5 g	Symptomatic improvement (WOMAC, VAS, Lequesne)	[106]
		Randomized double-blind placebo-controlled study	6 months	8 g	Symptomatic improvement (WOMAC, VAS)	[107]
	non-Osteoarthritis	Randomized double-blind placebo-controlled study	3 months	5 g	Reduction of exercise-induced knee pain	[111]
		Randomized double-blind placebo-controlled study	6 months	10 g	Reduction of join pain at rest and during activity	[112]
		Randomized double-blind placebo-controlled study	3 months	10 g	No significant differences vs. Placebo	[113]

Abbreviatures: Western Ontario and McMaster Universities Osteoarthritis Index (WOMAC); Visual Analogue Scale (VAS).

## Data Availability

Not applicable.
